# Assessing Mixture Effects of Cereulide and Deoxynivalenol on Intestinal Barrier Integrity and Uptake in Differentiated Human Caco-2 Cells

**DOI:** 10.3390/toxins13030189

**Published:** 2021-03-04

**Authors:** Julia Beisl, Elisabeth Varga, Dominik Braun, Benedikt Warth, Monika Ehling-Schulz, Giorgia Del Favero, Doris Marko

**Affiliations:** 1 Department of Food Chemistry and Toxicology, University of Vienna, Währinger Straße 38, 1090 Vienna, Austria; julia.beisl@univie.ac.at (J.B.); elisabeth.varga@univie.ac.at (E.V.); dominik.braun@univie.ac.at (D.B.); benedikt.warth@univie.ac.at (B.W.); giorgia.del.favero@univie.ac.at (G.D.F.); 2 Institute of Microbiology, Department of Pathobiology, University of Veterinary Medicine, Veterinärplatz 1, 1210 Vienna, Austria; Monika.Ehling-Schulz@vetmeduni.ac.at; 3 Core Facility Multimodal Imaging, Faculty of Chemistry, University of Vienna, Währinger Straße 38, 1090 Vienna, Austria

**Keywords:** *Bacillus cereus*, *Fusarium*, LC-MS/MS, paracellular passage, intestinal epithelium, food safety, bioavailability

## Abstract

The human intestine is regularly exposed to ingested food contaminants, such as fungal and bacterial toxins, which have been described to co-occur in a mixed diet. Thus, it is of utmost importance to understand possible interactions between contaminants of different origin. Hence, we investigated the single and combined effects of one of the most abundant mycotoxins, deoxynivalenol (DON; 0.1 to 10 µg/mL), and the bacterial toxin cereulide (CER; 1 to 100 ng/mL) on differentiated human Caco-2 (C2BBe1) cells cultured in a transwell system. We tested the capacity of the two toxins to alter the intestinal integrity and further investigated the uptake of both compounds and the formation of selected DON metabolites. CER alone (10 and 100 ng/mL) and in combination with DON (10 ng/mL CER with 1 µg/mL DON) was found to alter the barrier function by increasing the transepithelial electrical resistance and the expression of the tight junction protein claudin-4. For the first time, DON-3-sulfate was identified as a metabolite of human intestinal cells in vitro. Moreover, co-incubation of CER and DON led to an altered ratio between DON and DON-3-sulfate. Hence, we conclude that co-exposure to CER and DON may alter the intestinal barrier function and biotransformation of intestinal cells.

## 1. Introduction

The intestinal barrier, including epithelial cells as well as mucus and the intestinal immune response, represents the first line of defense against various ingested contaminants, such as mycotoxins and bacterial toxins. Abia et al. [1] recently reported the presence of several mycotoxins including one of the most prevalent toxic fungal metabolites, deoxynivalenol (DON, vomitoxin), together with the bacterial toxin cereulide (CER) in maize-based porridge. DON, produced by *Fusarium* species, contaminates grains such as wheat, maize or oats and their processed products to a variable extent [2,3,4]. CER, a metabolite of *Bacillus cereus* strains with a certain genotype [5], was detected in raw and boiled rice or pasta as well as dairy products [6,7,8]. Therefore, both toxins may co-occur in staple foods as recently described [1] or may be consumed simultaneously with a mixed diet, hence making combinatory exposure scenarios plausible.

Acute DON intoxication causes emesis; however, in chronic low doses, DON negatively impacts the intestinal barrier integrity. Reduced transepithelial electrical resistance (TEER) as a measure of barrier integrity was repeatedly reported in vitro but also the tight junction physiology can be altered by DON (for a detailed review, see [9]). For instance, after incubation with DON, the tight junction protein claudin-4 (CLD-4) was diminished in the human intestinal Caco-2 cell line [10,11]. Furthermore, altered nutrient absorption [12] as well as indications of intestinal inflammation [13,14,15] were reported in human intestinal epithelial cells after DON incubation. Recently, the importance of intestinal mucus in the toxicological evaluation of mycotoxins has been recognized [16]. Wan et al. [17] reported DON to alter the expression of the mucus proteins mucin 5AC and mucin 5B, which cover the intestinal epithelium as a part of the physical gut barrier. Furthermore, DON is known to be rapidly absorbed and excreted via urine [18,19,20] and DON is subject to phase II metabolism, with DON-glucuronides and DON-sulfates as biotransformation products that can be quantified in human urine as biomarkers of dietary exposure [21]. 

CER, a cyclic dodecadepsipeptide [D-O-Leu-D-Ala-L-O-Val-L-Val]_3_, causes nausea and vomiting as symptoms of acute intoxications [22,23]. Moreover, necrosis of colon mucosa has been reported in a fatal case of CER poisoning [24]. Until now, little is known about the effects of CER on human intestinal cells, including reports on cytotoxicity [25,26], alterations of the inflammatory response [26], mitochondrial dysfunction [27] and reduction of proteins involved in energy management and H_2_O_2_ detoxification [25]. Recent reports on the uptake and distribution of CER in piglets revealed that the toxin is accumulated in intestinal tissue to a great extent [28].

Increased paracellular permeability and a decrease in different tight junction proteins have been connected to increased pro-inflammatory cytokines, such as interleukin-1β and tumor necrosis factor-α [29,30]. Furthermore, Capaldo et al. [31] reported that pro-inflammatory cytokines decrease the integration of the tight junction protein CLD-4, thus leading to epithelial barrier dysfunction. As CER was previously shown to change the inflammatory response [26], we hypothesized that CER, alone or in combination with DON, may alter the complex interplay of the intestinal epithelial barrier including barrier integrity and toxin metabolism.

The aim of this study was therefore (I) to investigate the effect of CER on the barrier integrity and gain first insights on the uptake of CER in a human intestinal cell model, and (II) to study potential interactions of physiologically relevant concentrations of CER and DON concerning barrier integrity, uptake and metabolism. The investigation was aimed at a deeper understanding of interactions between bacterial and fungal toxins and their possible effects on pathologies of the human gut.

## 2. Results

### 2.1. TEER

**Figure 1:**
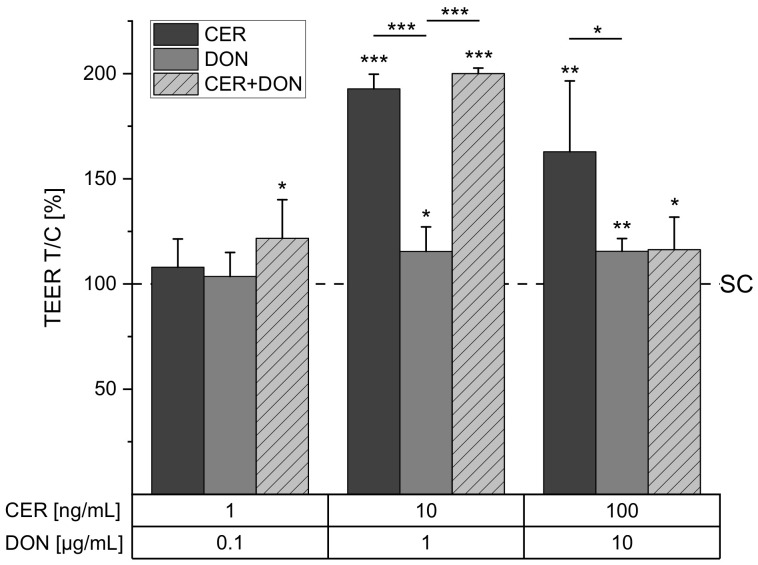
Transepithelial electrical resistance (TEER) of differentiated C2BBe1 cells after 24 h incubation with cereulide (CER, dark grey), deoxynivalenol (DON, grey) or a combination of CER and DON (light grey, dashed) or solvent control (SC, 1% DMSO, 1% H_2_O, dashed line). Results are depicted as mean + standard deviation of at least 5 biological replicates. and are normalized to the solvent control (dashed line). Significant differences between the solvent control and the incubation conditions were calculated by one-sample Student’s *t*-test. Significances between single substances and the combination were calculated by two-sample Student’s *t*-test. All significances are indicated with “*” (*p* < 0.05), “**” (*p* < 0.01) or “***” (*p* < 0.001).

Transepithelial electrical resistance was investigated as a measure for intestinal integrity. While in the lowest concentration of 1 ng/mL CER had no impact on TEER values, incubation with 10 and 100 ng/mL CER significantly increased TEER values to 193 ± 7% and 163 ± 34%, respectively (Figure 1). DON on the other hand showed only a slight induction of TEER to 116 ± 12% at 1 µg/mL DON and 116 ± 6% at 10 µg/mL DON. The combination of the two substances increased TEER already in the lowest applied concentration of 1 ng/mL CER together with 0.1 µg/mL DON. The combination of 10 ng/mL CER + 1 µg/mL DON further increased the TEER to 200 ± 3%, whereas this effect was reversed to 116 ± 16% at the highest combination (100 ng/mL CER + 10 µg/mL DON).

### 2.2. Paracellular Permeability: Lucifer Yellow (LY)

**Figure 2:**
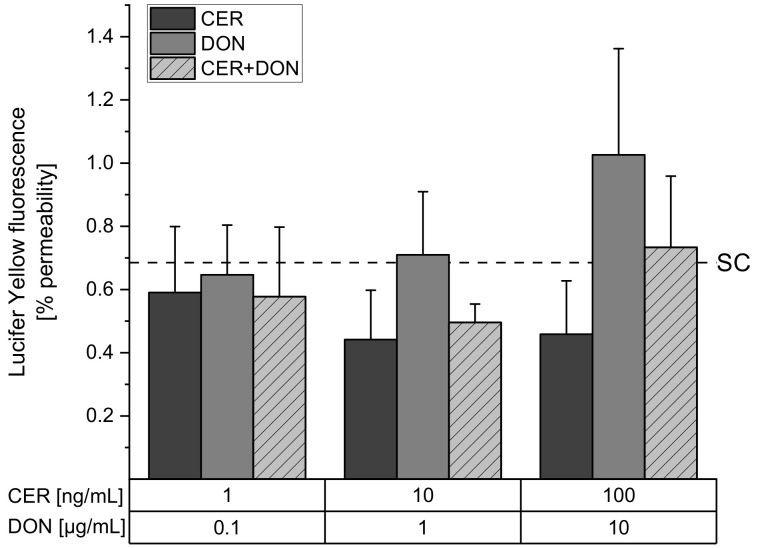
Lucifer Yellow fluorescence intensity in the basolateral compartment after 24 h incubation with CER (dark grey), DON (grey) or a combination of CER and DON (light grey, dashed) or solvent control (SC, 1% DMSO, 1% H_2_O, dashed line). Results are depicted as mean + standard deviation of 3 biological replicates.

Subsequently, barrier integrity was investigated by the paracellular passage of the small hydrophilic compound Lucifer Yellow. When the permeability is impaired, more dye can cross the membrane, hence TEER and paracellular permeability are negatively correlated. In the solvent control, 0.69 ± 0.26% of the dye was able to cross the intestinal cell monolayer and was therefore accepted as tight (Figure 2). Even though we see a trend that CER decreases the passage of LY through the monolayer and DON shows a tendency to increase the transit of the dye, no significant effects are visible. When comparing the results of the Lucifer Yellow assay to the TEER measurements, it seems as if LY reflects the observed TEER values, though not significantly.

### 2.3. Immunofluorescence

**Figure 3:**
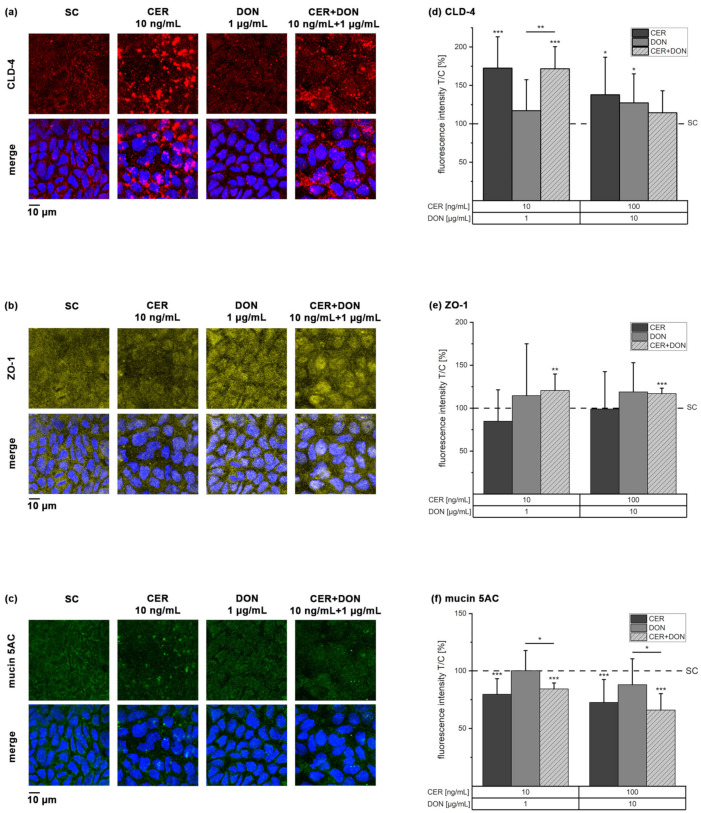
Immunofluorescence staining and quantitative analysis of claudin-4 (**a**,**d**), zona occludens 1 (**b**,**e**) and mucin 5AC (**c, f**) after 24 h incubation of differentiated Caco-2 cells with CER, DON, their combination or solvent control (SC). The microscopy panels (**a–c**) depict the single channel pictures in the upper row and the merge pictures with the nuclei (stained with DAPI) in the lower row. The bar charts (**d–f**) represent the quantitative analysis of the fluorescence intensities after incubation with CER (dark grey), DON (grey) and a combination of both (light grey, dashed). Results are presented as mean + standard deviation of 3 biological replicates including in total at least 9 optical fields and are normalized to the solvent control (dashed line). Significant differences between the solvent control and the incubation conditions were calculated by one-sample Student’s *t*-test. Significances between single substances and the combination were calculated by two-sample Student’s *t*-test. All significances are indicated with “*” (*p* < 0.05), “**” (*p* < 0.01) or “***” (*p* < 0.001).

To further investigate the origin of the changes in barrier function, immunofluorescence staining of the tight junction protein claudin-4 (CLD-4) and the scaffold protein zona occludens 1 (ZO-1) was performed. The selection of the tight junction proteins is based on recurrent literature reports of DON affecting CLD-4 and ZO-1 [9]. Incubation with 10 and 100 ng/mL CER significantly enhanced immunolocalization of CLD-4 (173 ± 41% and 138 ± 49% in comparison to the solvent control, Figure 3a,d). Furthermore, DON increased the CLD-4-associated fluorescence signal to 127 ± 38% in the highest applied concentration of 10 µg/mL DON. The combination of 10 ng/mL CER with 1 µg/mL DON likewise showed a significant increase of CLD-4 protein to 172 ± 29%, therefore reflecting the measured TEER values. However, this combination significantly differs from the respective effect of DON alone, suggesting the effect to be dominated by CER. ZO-1 on the other hand was significantly enhanced only by the tested substance in combinations but not by the single compounds (Figure 3b,e).

As the mucus of the intestine may also play an important role concerning barrier integrity, we investigated the effect of CER and DON on the expression of mucin 5AC. While DON alone had no significant effect, CER alone decreased mucin 5AC fluorescence intensity to 80 ± 14% and 73 ± 20% (10 and 100 ng/mL CER; Figure 3c,f). In combination with 1 and 10 µg/mL DON, mucin 5AC was significantly decreased to 84 ± 5% and 66 ± 14% compared to the solvent control. As with CLD-4, mucin 5AC is significantly reduced in the combinations compared to DON alone, likewise suggesting CER to be the major impact compound.

### 2.4. LC-MS/MS

**Figure 4:**
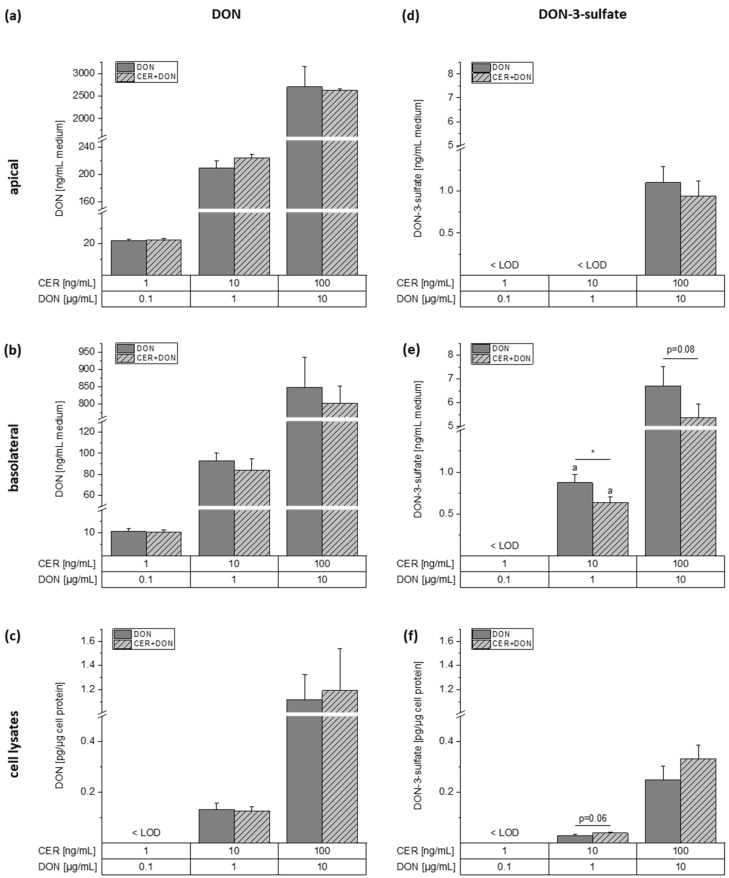
Concentrations of DON in the apical (**a**) and basolateral (**b**) medium as well as the cell lysates (**c**) and concentrations of DON-3-sulfate in the apical (**d**) and basolateral (**e**) medium and the cell lysates (**f**) after incubation of differentiated Caco-2 (C2BBe1) cells in transwell plates for 24 h with DON or DON together with CER. Grey bars represent incubations with DON and light grey, dashed bars represent incubations with the combination. Results are depicted as mean + standard deviation of 3 biological replicates. The letter “a” indicates that at least one measurement was < limit of quantification (LOQ). Differences between the treatment conditions were tested by two-sample Student’s *t*-test. Significances are indicated with “*” (*p* < 0.05).

Changes in intestinal integrity may lead to an altered uptake of contaminants, such as fungal or bacterial toxins. To study whether the combined exposure to CER and DON leads to an increased or decreased uptake, LC-MS/MS analysis of both toxins was performed. Furthermore, DON-3-sulfate (DON-3-S), DON-15-sulfate and DON-3-glucuronide were included in the analysis to investigate a possible effect of CER on the phase II metabolism of DON. Due to a lack of an authentic DON-15-glucuronide analytical standard, no quantitative assessment would have been possible. However, no peaks were observed at the expected retention time, and consequently, we concluded that DON-15-glucuronide is not a major metabolite of the tested cell line. As parent compound, DON was detected in all compartments and incubation conditions, except the cell lysates treated with the lowest toxin concentration (Figure 4a–c). In the apical compartment, no difference between the individual DON treatment and the combination with CER was observed. According to our analysis, DON translocated from the apical to the basolateral compartment in a similar extent in all applied concentrations even though a non-significant trend of a CER induced reduction of DON in the basolateral medium may be apparent. Moreover, we were able to detect DON in the cell lysates without significant difference between the single substance and the combination treatment.

Additionally, DON-3-S was detected in all analyzed compartments (Figure 4d–f). To our knowledge, this is the first report of the generation of DON-3-S by differentiated Caco-2 cells. Interestingly, in the apical medium, DON-3-S was merely detected after incubation with the highest concentration of DON (10 µg/mL) resulting in 1.1 ± 0.2 ng/mL and 0.9 ± 0.2 ng/mL DON-3-S for DON alone and in combination with 100 ng/mL CER, respectively. On the contrary, DON-3-S was detected in the two highest treatment conditions of the basolateral medium. Strikingly, approximately 6-times higher DON-3-S concentrations were found in the basolateral compared to the apical medium, suggesting an increased release of DON-3-S towards the basolateral side. After incubation with 1 µg/mL DON alone or combined with 10 ng/mL CER, 0.9 ± 0.1 and 0.6 ± 0.1 ng/mL DON-3-S were detected leading to a significant decrease of DON-3-S in the presence of CER. The highest concentration of DON and the combination shows the same but non-significant trend leading to DON-3-S concentrations of 6.7 ± 0.8 and 5.4 ± 0.6 ng/mL medium. DON-3-S was detected in the cell lysates of the two highest concentrations as well. While 1 µg/mL DON and the combination with 10 ng/mL CER led to DON-3-S concentrations of 0.028 ± 0.004 and 0.038 ± 0.005 pg/µg cell protein, the highest treatment concentration of 10 µg/mL DON as a single compound or together with 100 ng/mL CER resulted in 0.25 ± 0.05 and 0.33 ± 0.06 pg/µg cell protein, respectively.

**Figure 5:**
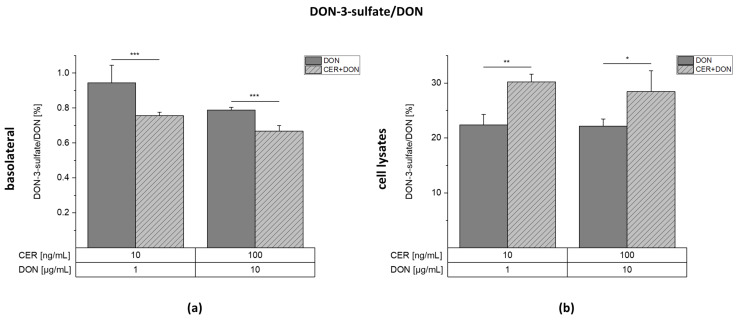
Ratio between detected DON-3-sulfate and DON in the basolateral medium (**a**) and the cell lysates (**b**) after incubation of differentiated Caco-2 cells in transwell plates for 24 h with DON or DON together with CER. Grey bars represent incubations with DON and light grey, dashed bars represent incubations with the combination. Results are depicted as mean + standard deviation of 3 biological replicates. Differences between the treatment conditions were tested by two-sample Student’s *t*-test. Significances are indicated with “*” (*p* < 0.05), “**” (*p* < 0.01) or “***” (*p* < 0.001).

Furthermore, a contrasting proportion between DON and DON-3-S concentrations appears to be present in the basolateral medium and the cell lysates of the two highest test concentrations (Figure 5). In the basolateral medium, roughly 0.9% of DON-3-S was detected in relation to DON. Contrary, in the cell lysates, the ratio of DON-3-S to DON is 20-30%. This suggests that more DON-3-S is present inside the cells in relation to DON than in the medium. Furthermore, CER affects the presented ratio distinctively, depending on the compartment. In the basolateral medium, the ratio of DON-3-S is significantly decreased from for instance 0.94 ± 0.10% in the samples incubated with 1 µg/mL DON to 0.76 ± 0.02% in the case of 1 µg/mL DON + 10 ng/mL CER. On the contrary, the ratio is significantly increased from 22.4 ± 1.9% (1 µg/mL DON) to 30.2 ± 1.4% (1 µg/mL DON + 10 ng/mL CER) in the cell lysates. Therefore, we conclude that the presence CER influences the metabolic behavior of the cells towards DON. 

DON-15-sulfate and DON-3-glucuronide were not detected in any sample. The limit of detection (LOD) and the limit of quantification (LOQ) were calculated based on the lowest standard, considering matrix effects and the dilution during sample preparation in the case of the medium. The LOD and LOQ for DON-15-sulfate were estimated to be 0.2 and 0.6 ng/mL in the incubation medium and 0.03 and 0.09 ng/mL in the lysate measurement solution. For DON-3-glucuronide, the LOD and LOQ were determined to be 0.7 and 2 ng/mL in the incubation medium as well as 0.1 and 0.4 ng/mL in the lysate measurement solution, respectively.

**Figure 6:**
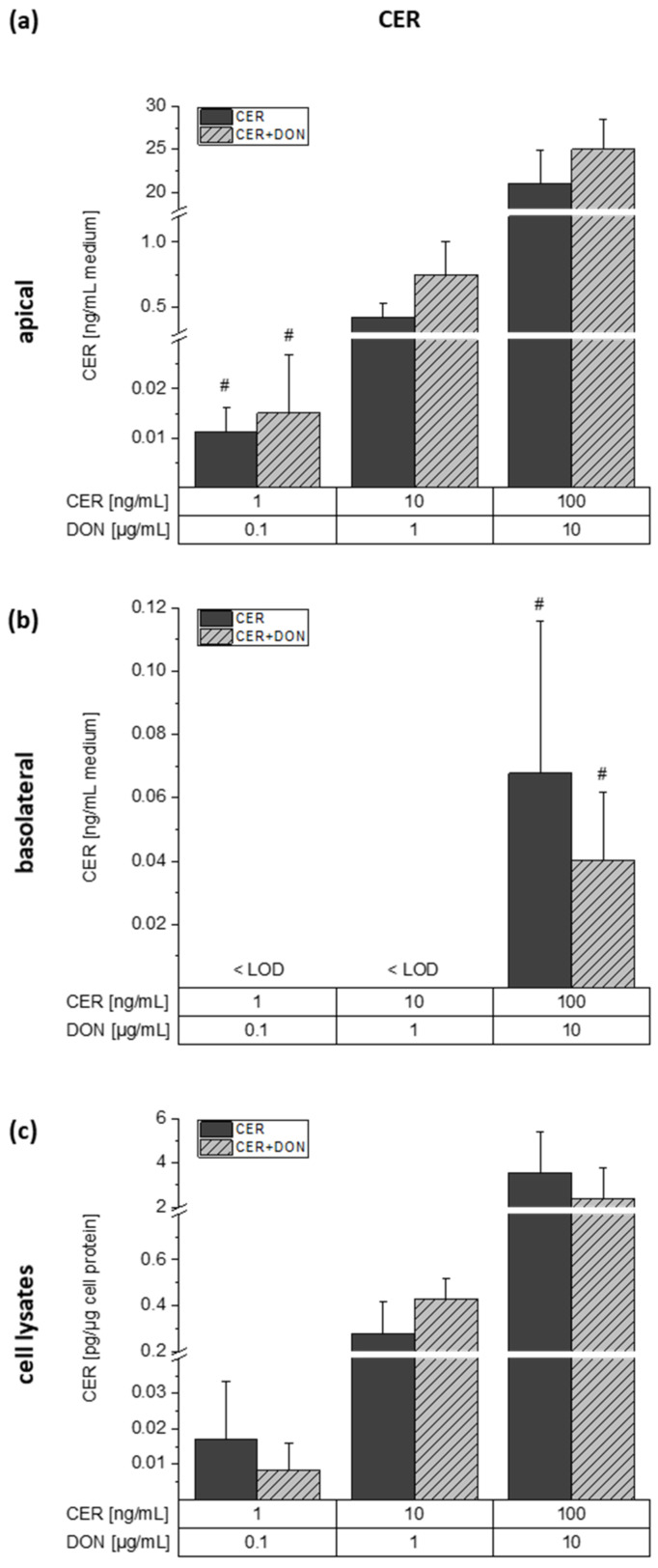
Concentrations of CER in the apical (**a**) and basolateral (**b**) medium as well as the cell lysates (**c**) after incubation of differentiated Caco-2 cells with CER or a combination of CER and DON for 24 h in transwell plates. Dark grey bars represent incubations with CER and light grey, dashed bars represent incubations with the combination. Results are depicted as mean + standard deviation of 3 biological replicates. Bars marked with “#” include concentrations calculated by one-point calibration. Differences between the treatment conditions were tested by two-sample Student’s *t*-test.

CER was likewise detected in all analyzed compartments (Figure 6). In the apical compartment, CER could be quantified in all treatment conditions with a slight trend toward higher CER recovery in the samples containing both CER and DON. In the basolateral compartment, CER was merely detected in the samples incubated with the highest toxin concentration of 100 ng/mL CER with or without 10 µg/mL DON in concentrations of 0.07 ± 0.05 ng/mL medium and 0.04 ± 0.02 ng/mL medium, respectively. While low amounts of CER were transported to the basolateral medium, CER was detected in the cell lysates of all samples in similar percentages. However, no significant differences between the single and combined incubations were observed.

## 3. Discussion

Humans are constantly exposed to a mixture of secondary metabolites of bacterial, fungal or plant origin. However, to our knowledge, this is the first report on effects of the bacterial toxin CER alone and in combination with the mycotoxin DON in a Caco-2 transwell model. In this manuscript, we present data concerning possible interactions on barrier integrity (Figure 1, Figure 2 and Figure 3) or even absorption and metabolism in human intestinal cells (Figure 4, Figure 5 and Figure 6). Our work revealed that the presence of CER increases both TEER (Figure 1) and the tight junction protein CLD-4 (Figure 3), suggesting an alteration of barrier function. As these effects were not only reported after incubations with CER as a single compound but also in the constant 1:100 combination with DON, CER may be the major impact compound in this context.

Concentration conditions were based on exposure data [1,2,3,7] and previous results [26] as risk assessment attempts are only available for mycotoxin mixtures [32] but not for the combination of *Fusarium* and *Bacillus cereus* toxins. As DON levels in cereals globally vary significantly between years and regions, the selected concentrations reflect a wide range of occurrence data [2,3,32]. Concerning CER, data on occurrence and exposure are scarce. Therefore, the applied concentrations are based on the occurrence in cooked rice and maize-fufu [1,7]. As previously reported, we assumed that the toxins were diluted in 1 L gastric fluid and are fully bio-accessible [26,33,34]. Furthermore, the applied ratio of 1:100 of CER:DON was selected based on their similar cytotoxic behavior after 24 h of incubation [26]. To avoid interpretation artefacts related to the onset of cytotoxicity, a minimum of ~80% viability was accepted for the applied incubation conditions, which was achieved for a broad concentration range after 24 h of incubation with CER and DON [26].

While, so far, toxicological research concerning the influence of CER on human intestinal cells mainly focused on cytotoxicity and mitochondria [25,27], the effects of DON on barrier integrity have been extensively studied (for review, see [9]). Twelve-hour and 24-h incubation did not show a significant reduction in TEER values in some laboratories [13,35], which is in line with the findings presented in the present study (Figure 1). On the contrary, prolonged incubation with DON was repeatedly reported to impair barrier integrity [9] and some authors reported decreased TEER values already after 24 h [11,34], which may be related to different culture conditions as previously reported [36]. In contrast, literature on substances increasing TEER, as reported in the present study by CER and the combination of CER and DON (Figure 1), is still scarce. For example, some *Lactobacillus* strains were found to increase TEER values in a Caco-2 cell model. Furthermore, the decrease of barrier integrity in the highest tested combination of DON and CER (Figure 1) may be related to the onset of cytotoxicity even though cell viability does not drop below 80% [26].

In addition to the investigation of barrier integrity, interest in the influence of mycotoxins on the intestinal mucus layer recently emerged, whereby data is still scarce [16]. Caco-2 cells have been described to possess mucus forming vacuoles in the cytoplasm and to express mucin 5AC [37]. Wan, Allen, Turner and El-Nezami [17] reported the upregulation of mucin 5AC after 48 h incubation with 2 µM DON in cell lysates of Caco-2 cells. In the present study, 24 h incubation with 1 and 10 µg/mL DON did not induce the same effect (Figure 3c,f). In addition to the different incubation times and conditions, a possible explanation for differences may be the difficulty to fix mucus with formaldehyde [16], thereby possibly partly losing mucins. In contrast to DON, CER and the mixture of CER and DON significantly reduced mucin 5AC protein content measured by fluorescence microscopy (Figure 3c,f). Therefore, we hypothesize that the secretion of mucus may be impaired by CER. Hence, the results gained from the mucin 5AC antibody staining (Figure 3c,f) represent the basis for further investigations concerning the involvement of CER in mucus pathologies.

DON was previously reported to decrease CLD-4 protein levels [11,33], which is not reflected in our results (Figure 3a,d), possibly due to differences in the test systems. The Caco-2 clone used in this manuscript, C2BBe1, is known for the improved morphological homogeneity and exclusive apical villin localization compared to the parental cell line (HTB-37) [38]. Therefore, differences in tight junction proteins cannot be excluded, as literature on the comparison of the two cell lines as well as information on the used clone is mostly lacking. Furthermore, we speculate that studies which did not identify decreased TEER values were also not investigating changes in claudin expression.

Additionally, DON was described to induce inflammation [13,26]. Together with the alterations in tight junction proteins, these effects are connected to the mitogen-associated protein kinase (MAPK) pathway representing a mechanism of action for DON toxicity [35,39]. Notably, CER significantly increased CLD-4 protein expression in our test system (Figure 3a,d). This effect persists even when the cells are co-incubated with DON (Figure 3a,d), hence raising the question how CER interacts with the tight junctions. Previous co-incubation studies of DON with, for instance, resveratrol [33] or galacto-oligosaccharides [40] demonstrated protective properties towards the destruction of tight junctions by DON via the modulation of the inflammatory response or the MAPK pathway. We previously reported diminished inflammatory properties of DON in the presence of CER [26]. Therefore, it is tempting to speculate that CER may interact with the activation of the MAPK pathway and may thus also influence tight junctions.

With regard to intestinal pathologies such as inflammatory bowel disease, several tight junction proteins including CLD-4 were described to be upregulated in ulcerative colitis associated colon carcinomas [41]. Furthermore, increased CLD-4 expression in colo-rectal cancer was described to be associated with an altered barrier function and disorganization of tight junctions [42]. Indeed, it remains to be uncovered how the distinct effects of CER and DON on different toxicological endpoints of the intestinal barrier integrity (Figure 1, Figure 2 and Figure 3) may potentiate or ameliorate each other and how they may be connected to inflammatory events.

Subsequently, we investigated the uptake of CER and DON and whether co-incubations of the toxins impact the uptake behavior of the cells (Figure 4, Figure 5 and Figure 6). Furthermore, by analyzing phase II metabolites of DON, we addressed the question of possible interactions concerning DON metabolism (Figure 4 and Figure 5). DON uptake in the intestine is driven by trans- and paracellular passage [34]. Therefore, it was likely to speculate that due to the observed increase of monolayer tightness after CER incubation (Figure 1), DON may be taken up to a lesser extent, which was not the case (Figure 4). While DON-3-S was recently reported to be produced by HepG2 liver cells [43], the present study demonstrates for the first time the formation of this metabolite in differentiated Caco-2 cells (Figure 4). Our data support the hypothesis that DON-3-S is preferentially excreted to the basolateral side of the transwell system (Figure 4). Thus, we conclude that intestinal cells may indeed contribute to the amount of DON-3-S present in human urine as reported previously [21]. Furthermore, CER seems to interact with the metabolism or secretion of DON-3-S as in the presence of CER significantly higher amounts of DON-3-S in relation to DON were detected in the cell lysates (Figure 5). Concerning CER, an accumulation especially in the intestine of piglets fed with CER was reported [28]. Our results reflect these findings, as we detected the highest concentrations of CER in the cell lysates (Figure 6). Nevertheless, CER was detected in the basolateral compartment to some extent (Figure 6) and may therefore reach the liver as well as other organs even if present in only low concentrations in food. 

## 4. Conclusions

In conclusion, we reported for the first time the effect of CER on Caco-2 cells in a transwell model. The impact of CER seems to be abundant in endpoints representing measures for intestinal integrity indicated by increased tightness of the intestinal barrier alone and in combination with DON. While we present data implying the accumulation of CER in intestinal tissue, CER has a limited impact on the uptake of DON. Nevertheless, our studies imply that CER may influence DON metabolism in intestinal cells. Co-exposure to CER and DON is quite likely to occur globally due to their prevalence in basic foodstuffs, hence raising the question how the toxins may influence pathophysiological processes. Based on our results and presented literature, effects of the two toxins on diseases such as inflammatory bowel syndrome, intestinal lesions or leaky gut syndrome cannot be excluded and should be addressed in future research. Additionally, further analysis including co-occurrence data of mycotoxins and bacterial toxins is necessary.

## 5. Materials and Methods

### 5.1. Chemicals and Consumables

DON was obtained from Romer Labs (Tulln, Austria) and CER was purified as previously described [28]. Cell culture media and supplements were obtained from Gibco® Life Technologies (Karlsruhe, Germany). KH*_2_*PO_4_, KCl, NaCl, Na_2_HPO_4_, D-glucose, HEPES, CaCl_2_, MgCl_2_, glycin, formaldehyde, sodium dodecyl sulfate (SDS), NaOH and Triton X-100 were purchased from Carl Roth (Karlsruhe, Germany) and Lucifer Yellow CH di-lithium salt from Santa Cruz Technologies (Dallas, TX, USA). For LC-MS/MS measurements, MS grade water was purchased from VWR (Fontenay-sous-Bois, France), acetonitrile (ACN) and methanol (MeOH) from Honeywell (Seelze, Germany). Acetic acid was obtained from Merck (Darmstadt, Germany) and ammonium acetate (both LC-MS grade) from Sigma Aldrich (Vienna, Austria). DON-3-sulfate, DON-15-sulfate and DON-3-glucuronide were kindly provided by Dr. Philipp Fruhmann (Technical University of Vienna) and synthesized as previously described [44].

### 5.2. Caco-2 (C2BBe1) Cells

All experiments were performed with the Caco-2 clone C2BBe1 (ATCC-CRL-2102), which was acquired from the American Type Culture Collection (ATCC; Manassas, VA, USA). C2BBe1 cells originate from a colorectal adenocarcinoma and are able to spontaneously differentiate into a polarized monolayer with an apical brush border. Cells were cultivated in Dulbecco’s Modified Eagle Medium (DMEM) supplemented with 10% (*v*/*v*) fetal calf serum (FCS), 100 U/mL penicillin, 100 µg/mL streptomycin, 1% (*v*/*v*) potassium pyruvate and 0.01 mg/mL transferrin under humidified conditions (37 °C, 5% CO_2_, 95% humidity). Cells were grown for 3–4 days before sub-cultivation at a confluency of 80% and seeded for experiments in TRANSWELL^®^ plates (12 well, 0.4 µm pore size; costar^®^, Corning Incorporated, Corning, NY, USA) or Falcon^®^ 8-well culture slides (Corning Incorporated, Corning, NY, USA) at a density of 85,000 cells/cm^2^. Prior to all experiments, cells were cultivated for seven days to obtain a partly differentiated, tight monolayer as previously reported [13,26,45]. Paracellular permeability was checked with the tracking dye Lucifer Yellow and the obtained results of ~1% represent a tight monolayer according to the manufacturers report (Sigma-Aldrich St. Louis, MO, USA; 2013). The medium was exchanged every 2–3 days. Passage numbers between 10 and 28 were used for the experiments and the cells were routinely checked for mycoplasma contamination.

### 5.3. Incubation Conditions 

CER and DON stock solutions were prepared in dimethylsulfoxide (DMSO) and water, respectively. Cells were incubated with 1–100 ng/mL CER (0.87–87 µM) and 0.1–10 µg/mL (0.34–34 µM) DON or co-incubated with the respective 1:100 combination with a final volume of 1% DMSO and 1% H_2_O. The chosen concentrations reflect the occurrence of CER and DON [1,2,46] and are discussed in Section 3. Medium containing both solvents served as a solvent control in all cell treatments. All the presented experiments include at least three biological replicates.

### 5.4. Transepithelial Electrical Resistance (TEER)

Epithelial integrity was examined with an Epithelial Voltohmmeter (EVOM2) coupled to a chopstick electrode pair (STX2, both World Precision Instruments, Sarasota, FL, USA) measuring TEER. To ensure reproducibility, plates were equilibrated at room temperature for 20 min prior to the TEER measurements as suggested [36]. Prior to the measurement, the chopstick electrodes were rinsed with ethanol and equilibrated at room temperature in medium. The mean value of three measurements per well was assessed and multiplied by the membrane area to obtain Ω × cm^2^. The TEER of the blank well was subtracted from the TEER of the treated wells and all samples are presented as percentage of the solvent control.

### 5.5. Paracellular Permeability: Lucifer Yellow

The small hydrophilic compound Lucifer Yellow (LY) was used to ensure the tightness of the monolayers and study changes in paracellular permeability of treated cells. For the assay, both compartments of the transwell system were washed with Hank’s balanced salt solution (HBSS) buffer containing 25 mM D-glucose, 20 mM HEPES, 1.25 mM CaCl_2_ and 0.5 mM MgCl_2_ at pH 7.4. Subsequently, 0.5 mL of a 0.1 mg/mL Lucifer Yellow CH di-lithium salt solution in HBSS buffer and 1.5 mL of pure HBSS buffer were added to the apical and basolateral compartment, respectively. After a 1 h incubation at 37 °C, fluorescence of the basolateral medium of all wells as well as pure LY solution were measured in triplicates (excitation: 485 nm, emission: 535 nm). The fluorescence of the sample wells was related to pure LY after subtraction of the blank (HBSS) and is reported as % permeability. In the solvent control, a LY permeability of < 1% was accepted for tight monolayers.

### 5.6. Immunofluorescence

To study the expression of the tight junction protein claudin-4 (CLD-4), zona occludens-1 (ZO-1) as well as mucin 5AC, an immunofluorescence staining with subsequent microscopic analysis was performed. Before cell seeding, 8-chamber culture slides (Falcon^®^, Corning Incorporated, Corning, NY, USA) were coated with 0.3 mg/mL matrigel in serum-free DMEM for 30 min at 37 °C. After the seven-day differentiation described in chapter 5.2, the cells were incubated with single toxins or combinations, namely 10 and 100 ng/mL CER and 1 and 10 µg/mL DON for 24 h. The two incubation concentrations were selected based on TEER and LY results. Immunofluorescence stainings were performed based on [47,48]. Briefly, cells were fixed with 3.7% formaldehyde (FA) in phosphate buffered saline (PBS) for 15 min followed by two washing steps with PBS and permeabilization with 0.2% Triton X-100 in PBS for 10 min. After rinsing with PBS, blocking was performed with 1% donkey serum in PBS for 1 h, directly followed by a 2 h incubation with the primary antibodies (anti-Claudin 4 antibody, ab53156, 1:500 dilution; anti-ZO-1 tight junction protein antibody, ab190085, 1:500 dilution; anti-Mucin 5AC antibody, ab77576, 1:250 dilution, from abcam, Cambridge, UK). Cells were rinsed thrice with washing buffer (0.05% Triton X-100 in PBS) and twice with PBS followed by a 1.5 h incubation with the respective fluorescently labelled secondary antibodies (donkey anti-rabbit IgG, Alexa Fluor 568, A10042 from Invitrogen, Thermo Fisher Scientific, Waltham, MA, USA; donkey anti-goat IgG, Alexa Fluor 647, 705-605-003 and donkey anti-mouse IgG, Alexa Fluor 488, 715-545-150 from Jackson ImmunoResearch Laboratories, West Grove, PA, USA; all in 1:750 dilution). The same washing procedure as before was applied to remove remaining unbound secondary antibody. A post-staining fixation was performed with 3.7% FA in PBS followed by masking of reactive sites with 100 mM glycine for 5 min. Slides were mounted with ROTI^®^Mount FluorCare DAPI mounting medium (Carl Roth, Karlsruhe, Germany). Image acquisition was performed with an LSM Zeiss 710 microscope equipped with an ELYRA PS.1 system with an AndoriXon 897 (EMCCD) camera and a Plan Apochromat 100X (1.46 NA) objective. Image analysis was performed with the ZEN 2012 SP3 (black) software. Microsoft Excel 2016 was used for further data evaluation. Experiments were performed in three biological replicates and four optical fields were randomly chosen per incubation condition resulting in at least 9 images/data point. 

### 5.7. Analytical Sample Preparation

After 24 h of incubation with CER, DON or a combination of both, 250 µL of apical and basolateral medium were transferred into an equal amount of pre-cooled ACN. The samples were stored at −20 °C for 1 h to precipitate proteins followed by 10 min centrifugation at 18,000× *g* at 4 °C. Thereafter, 450 µL of the supernatant were further diluted with an equal volume of water, transferred into 1.5 mL glass vials and stored at −80 °C until LC-MS/MS analysis. To obtain cell lysates, both the apical and the basolateral compartment were washed with PBS followed by the removal of the membrane from the transwell insert. The membranes containing the cell monolayers were transferred into tubes preloaded with 0.6 mL of ice-cold quenching solution (40% ACN, 40% MeOH, 20% H_2_O) and immediately flash-frozen in liquid nitrogen. For complete cell lysis, the samples were thawed in iced water, vortexed for 30 s, sonicated in iced water for 10 min and flash frozen in liquid N_2_ three times in total. After thawing the samples on ice, centrifugation at 15,000× *g* for 15 min at 4 °C enabled separation of the precipitated proteins. The supernatant was transferred into a fresh Eppendorf tube, evaporated to dryness in a vacuum concentrator at 4 °C (Labconco, MO, USA) and stored at −80 °C until reconstitution and analysis.

The protein content of the protein pellets was determined with a Bicinchoninic Acid (BCA) Protein Assay Kit (BCA1 and B9643 from Sigma-Aldrich, St. Louis, MO, USA) based on the manufacturer’s instructions. Briefly, protein pellets were dissolved in 40 µL of a 5% SDS solution in 0.1 N NaOH, vortexed, sonicated and further diluted with 60 µL of deionized water. The BCA working reagent was prepared by adding 0.2 mL of a 4% copper(II)sulfate pentahydrate solution to 10 mL of BCA solution. Six standards ranging from 0 to 1 mg/mL were prepared in the same buffer as the samples. Subsequently, 45 µL of dissolved protein or standard were incubated with 360 µL of BCA working reagent for 30 min at 37 °C. All samples had to be further diluted 1:1 prior to the absorbance measurement of duplicates at 570 nm in a Victor V3 (Perkin Elmer, Waltham, MA, USA) plate reader. Protein concentrations were calculated with the protein standard curve. The lysate supernatants containing the toxins of interest were reconstituted in 20% MeOH standardized to their protein content and stored at −80 °C until analysis. 

### 5.8. LC-MS/MS 

Sample analysis was performed with a 1290 Infinity II LC System (Agilent Technologies, Waldbronn, Germany) coupled to a QTrap 6500+ LC-MS/MS system (AB Sciex, Redwood City, CA, USA). For the analysis of DON and its biotransformation products and CER, two tailored methods were applied. This ensured highest sensitivities for CER and DON and its metabolites which required the presence or absence of ammonium acetate in the eluents. The LC-MS/MS system was operated by Analyst 1.7.0 software, whereas data analysis and evaluation were performed with Sciex OS 1.5.0 and Microsoft Excel 2016. Results are expressed as detected analyte in ng/mL medium for basolateral and apical medium and pg/µg cell protein for cell lysate samples.

#### 5.8.1. Determination of DON and Key Metabolites

The chromatographic separation of DON and its phase II metabolites was based on a previously published method [21]. Briefly, a Kinetex^®^ Biphenyl 100 Å column (150 × 3.0 mm, 2.6 µm, Phenomenex, Aschaffenburg, Germany) equipped with a guard column of the same type was operated at 40 °C. The mobile phases consisted of H_2_O/10% MeOH/0.05% acetic acid (eluent A) and MeOH/0.05% acetic acid (eluent B). A flow rate of 0.4 mL/min was applied. After an initial one-minute period of pure eluent A, the percentage of eluent B was raised linearly to 16% within 9 min, further to 95% B within 2 min and was held constant for 4 min. To reach the initial conditions, a steep gradient was employed within 0.1 min followed by a 3 min hold time at 100% eluent A, resulting in a total runtime of 19 min. For neat-solvent standards and lysate samples, the default injection volume of 5 µL was applied, whereas the injection volume of the medium samples was reduced to 3 µL to avoid peak distortion. The QTrap 6500+ was operated in negative electrospray ionization mode with a Turbo Spray IonDrive ion source applying the following parameters: curtain gas (CUR, nitrogen) 35 psi (241 kPa), collision gas (CAD, nitrogen) high, ion spray voltage (IS) −4500 V, temperature 450 °C, sheath gas (GS1) and drying gas (GS2) 60 psi (414 kPa, zero grade air). DON, DON-3-sulfate, DON-15-sulfate and DON-3-glucuronide were analyzed in the scheduled multiple reaction monitoring (MRM) mode. MRM parameters are reported in Table 1. DON-15-glucuronide could not be quantified as no reference standard was available. The polar metabolites of DON are known to be prone to retention time shifts. Therefore, standards in neat solvent were measured after each set (one replicate of apical and basolateral medium and lysate) to ensure correct identification.

#### 5.8.2. Determination of CER

The chromatographic separation of CER was performed with a ZORBAX Eclipse Plus C18 Rapid Resolution High Definition UHPLC column (2.1 × 50 mm, 1.8 µm, Agilent Technologies, Waldbronn, Germany) equipped with a SecurityGuard™ ULTRA Cartridges UHPLC C18 guard column (2.1 mm, Phenomenex, Aschaffenburg, Germany) at 40 °C. The mobile phases were composed of H_2_O/10 methanol/0.05% acetic acid (eluent A) and methanol/0.05% acetic acid/1 mM NH_4_Ac (eluent B) operated with a flow rate of 0.4 mL/min. Starting with a 0.5 min period of 90% eluent B, a linear gradient was applied raising eluent B from 90% to 100% in 2 min followed by a holding period of 1.5 min at 100% eluent B. To return to the initial conditions, a sharp gradient was applied within 0.1 min followed by a subsequent hold time of 0.9 min resulting in a total run time of 5 min. An injection volume of 5 µL was applied for all samples. The QTrap 6500+ system was operated in positive electrospray ionization mode with the same ion source and source parameters as for the detection of DON (Section 5.8.1). CER was acquired in the MRM mode and MRM parameters are reported in Table 1. CER data was evaluated by applying bracketing standard curve calculation and for the lowest concentrations of CER one-point calibration was performed.

### 5.9. Statistical Analysis.

Statistical analysis and graphical illustration were performed in OriginPro 2018G. To eliminate outliers and test for normal distribution of the data, the Nalimov outlier test and the Shapiro–Wilk normality test were employed on all data sets, respectively. One sample Student’s *t*-tests were performed to evaluate significant differences between the test conditions and the respective solvent control. To analyze differences between a single substance and the respective mixtures, two-sample Student’s *t*-tests were performed. The following p values were applied in all statistical analyses: * *p* < 0.05; ** *p* < 0.01; *** *p* < 0.001.

Mathematical models such as the “Combination Index Theorem” [49] or the “Independent Joint Action” [49,50] could not be applied as the experimental data did not fulfil the recommended conditions for modelling.

## Figures and Tables

**Table 1:** Multiple reaction monitoring (MRM) parameters for the determination of deoxynivalenol (DON), some of its key metabolites and cereulide (CER).

Analyte	Retention Time (min)	Precursor Ion (*m/z*)	Declustering Potential DP (V)	Product Ions ^a^ (*m/z*)	Collision Energy CE ^a^ (V)	Cell Exit Potential CXP ^a^ (V)	Entrance Potential EP (V)	Dwell Time (ms)	Ion Ratio Qualifier: Quantifier
DON	10.1	355.1	−50	265.2/59.2	−24/−36	−13/−9	−10	23	3.20
DON-3-sulfate	8.5–9.4 ^b^	375.0	−125	345.0/97.0	−36/−28	−21/−11	−10	36	0.35
DON-15-sulfate	8.1–8.9	375.0	−110	97.1/163.1	−38/−50	−9	−10	36	0.11
DON-3-glucuronide	9.5–9.7	471.1	−60	113.0/175.1	−35/−40	−12	−10	23	0.63
CER	2.0	1170.5	26	357.2/499.3	87/79	22/30	10	50	0.93

^a^ quantifier/qualifier, ^b^ sensitive to measurement conditions.

## Data Availability

Data is contained within the article.

## References

[1] Abia W.A., Warth B., Ezekiel C.N., Sarkanj B., Turner P.C., Marko D., Krska R., Sulyok M. (2017). Uncommon toxic microbial metabolite patterns in traditionally home-processed maize dish (fufu) consumed in rural Cameroon. Food Chem. Toxicol. Int. J. Publ. Br. Ind. Biol. Res. Assoc..

[2] Mishra S., Srivastava S., Dewangan J., Divakar A., Kumar Rath S. (2019). Global occurrence of deoxynivalenol in food commodities and exposure risk assessment in humans in the last decade: A survey. Crit. Rev. Food Sci. Nutr..

[3] Palumbo R., Crisci A., Venâncio A., Cortiñas Abrahantes J., Dorne J.-L., Battilani P., Toscano P. (2020). Occurrence and Co-Occurrence of Mycotoxins in Cereal-Based Feed and Food. Microorganisms.

[4] Spanic V., Katanic Z., Sulyok M., Krska R., Puskas K., Vida G., Drezner G., Šarkanj B. (2020). Multiple Fungal Metabolites Including Mycotoxins in Naturally Infected and *Fusarium*-Inoculated Wheat Samples. Microorganisms.

[5] Ehling-Schulz M., Svensson B., Guinebretiere M.H., Lindback T., Andersson M., Schulz A., Fricker M., Christiansson A., Granum P.E., Martlbauer E. (2005). Emetic toxin formation of *Bacillus cereus* is restricted to a single evolutionary lineage of closely related strains. Microbiology.

[6] Messelhausser U., Frenzel E., Blochinger C., Zucker R., Kampf P., Ehling-Schulz M. (2014). Emetic *Bacillus cereus* are more volatile than thought: Recent foodborne outbreaks and prevalence studies in Bavaria (2007–2013). Biomed. Res. Int..

[7] Delbrassinne L., Andjelkovic M., Dierick K., Denayer S., Mahillon J., Van Loco J. (2012). Prevalence and levels of *Bacillus cereus* emetic toxin in rice dishes randomly collected from restaurants and comparison with the levels measured in a recent foodborne outbreak. Foodborne Pathog. Dis..

[8] Dierick K., Van Coillie E., Swiecicka I., Meyfroidt G., Devlieger H., Meulemans A., Hoedemaekers G., Fourie L., Heyndrickx M., Mahillon J. (2005). Fatal family outbreak of *Bacillus cereus*-associated food poisoning. J. Clin. Microbiol..

[9] Akbari P., Braber S., Varasteh S., Alizadeh A., Garssen J., Fink-Gremmels J. (2017). The intestinal barrier as an emerging target in the toxicological assessment of mycotoxins. Arch. Toxicol..

[10] Pinton P., Nougayrede J.P., Del Rio J.C., Moreno C., Marin D.E., Ferrier L., Bracarense A.P., Kolf-Clauw M., Oswald I.P. (2009). The food contaminant deoxynivalenol, decreases intestinal barrier permeability and reduces claudin expression. Toxicol. Appl. Pharmacol..

[11] Akbari P., Braber S., Gremmels H., Koelink P.J., Verheijden K.A., Garssen J., Fink-Gremmels J. (2014). Deoxynivalenol: A trigger for intestinal integrity breakdown. Faseb J. Off. Publ. Fed. Am. Soc. Exp. Biol..

[12] Maresca M., Mahfoud R., Garmy N., Fantini J. (2002). The mycotoxin deoxynivalenol affects nutrient absorption in human intestinal epithelial cells. J. Nutr..

[13] Maresca M., Yahi N., Younes-Sakr L., Boyron M., Caporiccio B., Fantini J. (2008). Both direct and indirect effects account for the pro-inflammatory activity of enteropathogenic mycotoxins on the human intestinal epithelium: Stimulation of interleukin-8 secretion, potentiation of interleukin-1beta effect and increase in the transepithelial passage of commensal bacteria. Toxicol. Appl. Pharmacol..

[14] Woelflingseder L., Gruber N., Adam G., Marko D. (2020). Pro-Inflammatory Effects of NX-3 Toxin Are Comparable to Deoxynivalenol and not Modulated by the Co-Occurring Pro-Oxidant Aurofusarin. Microorganisms.

[15] Kadota T., Furusawa H., Hirano S., Tajima O., Kamata Y., Sugita-Konishi Y. (2013). Comparative study of deoxynivalenol, 3-acetyldeoxynivalenol, and 15-acetyldeoxynivalenol on intestinal transport and IL-8 secretion in the human cell line Caco-2. Toxicol. Vitr. Int. J. Publ. Assoc. Bibra.

[16] Robert H., Payros D., Pinton P., Theodorou V., Mercier-Bonin M., Oswald I.P. (2017). Impact of mycotoxins on the intestine: Are mucus and microbiota new targets?. J. Toxicol. Environ. Health. Part B Crit. Rev..

[17] Wan L.Y., Allen K.J., Turner P.C., El-Nezami H. (2014). Modulation of mucin mRNA (MUC5AC and MUC5B) expression and protein production and secretion in Caco-2/HT29-MTX co-cultures following exposure to individual and combined *Fusarium* mycotoxins. Toxicol. Sci. Off. J. Soc. Toxicol..

[18] Vidal A., Claeys L., Mengelers M., Vanhoorne V., Vervaet C., Huybrechts B., De Saeger S., De Boevre M. (2018). Humans significantly metabolize and excrete the mycotoxin deoxynivalenol and its modified form deoxynivalenol-3-glucoside within 24 hours. Sci. Rep..

[19] Warth B., Sulyok M., Berthiller F., Schuhmacher R., Krska R. (2013). New insights into the human metabolism of the *Fusarium* mycotoxins deoxynivalenol and zearalenone. Toxicol. Lett..

[20] Turner P.C., Hopton R.P., White K.L., Fisher J., Cade J.E., Wild C.P. (2011). Assessment of deoxynivalenol metabolite profiles in UK adults. Food Chem. Toxicol. Int. J. Publ. Br. Ind. Biol. Res. Assoc..

[21] Warth B., Del Favero G., Wiesenberger G., Puntscher H., Woelflingseder L., Fruhmann P., Sarkanj B., Krska R., Schuhmacher R., Adam G. (2016). Identification of a novel human deoxynivalenol metabolite enhancing proliferation of intestinal and urinary bladder cells. Sci. Rep..

[22] Ehling-Schulz M., Lereclus D., Koehler T.M. (2019). The *Bacillus cereus* Group: *Bacillus* Species with Pathogenic Potential. Microbiol. Spectr..

[23] Schoeni J.L., Wong A.C. (2005). *Bacillus cereus* food poisoning and its toxins. J. Food Prot..

[24] Naranjo M., Denayer S., Botteldoorn N., Delbrassinne L., Veys J., Waegenaere J., Sirtaine N., Driesen R.B., Sipido K.R., Mahillon J. (2011). Sudden death of a young adult associated with *Bacillus cereus* food poisoning. J. Clin. Microbiol..

[25] Rajkovic A., Grootaert C., Butorac A., Cucu T., De Meulenaer B., van Camp J., Bracke M., Uyttendaele M., Bacun-Druzina V., Cindric M. (2014). Sub-emetic toxicity of *Bacillus cereus* toxin cereulide on cultured human enterocyte-like Caco-2 cells. Toxins.

[26] Beisl J., Pahlke G., Abeln H., Ehling-Schulz M., Del Favero G., Varga E., Warth B., Sulyok M., Abia W., Ezekiel C.N. (2020). Combinatory effects of cereulide and deoxynivalenol on *in vitro* cell viability and inflammation of human Caco-2 cells. Arch. Toxicol..

[27] Decleer M., Jovanovic J., Vakula A., Udovicki B., Agoua R.E.K., Madder A., De Saeger S., Rajkovic A. (2018). Oxygen Consumption Rate Analysis of Mitochondrial Dysfunction Caused by *Bacillus cereus* Cereulide in Caco-2 and HepG2 Cells. Toxins.

[28] Bauer T., Sipos W., Stark T.D., Käser T., Knecht C., Brunthaler R., Saalmüller A., Hofmann T., Ehling-Schulz M. (2018). First Insights Into Within Host Translocation of the *Bacillus cereus* Toxin Cereulide Using a Porcine Model. Front. Microbiol..

[29] Al-Sadi R.M., Ma T.Y. (2007). IL-1β Causes an Increase in Intestinal Epithelial Tight Junction Permeability. J. Immunol..

[30] Ma T.Y., Iwamoto G.K., Hoa N.T., Akotia V., Pedram A., Boivin M.A., Said H.M. (2004). TNF-α-induced increase in intestinal epithelial tight junction permeability requires NF-κB activation. Am. J. Physiol. Gastrointest. Liver Physiol..

[31] Capaldo C.T., Farkas A.E., Hilgarth R.S., Krug S.M., Wolf M.F., Benedik J.K., Fromm M., Koval M., Parkos C., Nusrat A. (2014). Proinflammatory cytokine-induced tight junction remodeling through dynamic self-assembly of claudins. Mol. Biol. Cell.

[32] Battilani P., Palumbo R., Giorni P., Dall’Asta C., Dellafiora L., Gkrillas A., Toscano P., Crisci A., Brera C., De Santis B. (2020). Mycotoxin mixtures in food and feed: Holistic, innovative, flexible risk assessment modelling approach. EFSA Supporting Publ..

[33] Ling K.H., Wan M.L., El-Nezami H., Wang M. (2016). Protective Capacity of Resveratrol, a Natural Polyphenolic Compound, against Deoxynivalenol-Induced Intestinal Barrier Dysfunction and Bacterial Translocation. Chem. Res. Toxicol..

[34] Sergent T., Parys M., Garsou S., Pussemier L., Schneider Y.J., Larondelle Y. (2006). Deoxynivalenol transport across human intestinal Caco-2 cells and its effects on cellular metabolism at realistic intestinal concentrations. Toxicol. Lett..

[35] Pinton P., Braicu C., Nougayrede J.P., Laffitte J., Taranu I., Oswald I.P. (2010). Deoxynivalenol impairs porcine intestinal barrier function and decreases the protein expression of claudin-4 through a mitogen-activated protein kinase-dependent mechanism. J. Nutr..

[36] Srinivasan B., Kolli A.R., Esch M.B., Abaci H.E., Shuler M.L., Hickman J.J. (2015). TEER measurement techniques for *in vitro* barrier model systems. J. Lab. Autom..

[37] Bu X.D., Li N., Tian X.Q., Huang P.L. (2011). Caco-2 and LS174T cell lines provide different models for studying mucin expression in colon cancer. Tissue Cell.

[38] ATCC C2BBe1 [Clone of Caco-2] (ATCC® CRL-2102™). Characteristics. https://www.lgcstandards-atcc.org/products/all/CRL-2102.aspx?geo_country=at#characteristics.

[39] Springler A., Hessenberger S., Schatzmayr G., Mayer E. (2016). Early Activation of MAPK p44/42 Is Partially Involved in DON-Induced Disruption of the Intestinal Barrier Function and Tight Junction Network. Toxins.

[40] Akbari P., Braber S., Alizadeh A., Verheijden K.A., Schoterman M.H., Kraneveld A.D., Garssen J., Fink-Gremmels J. (2015). Galacto-oligosaccharides Protect the Intestinal Barrier by Maintaining the Tight Junction Network and Modulating the Inflammatory Responses after a Challenge with the Mycotoxin Deoxynivalenol in Human Caco-2 Cell Monolayers and B6C3F1 Mice. J. Nutr..

[41] Mees S.T., Mennigen R., Spieker T., Rijcken E., Senninger N., Haier J., Bruewer M. (2009). Expression of tight and adherens junction proteins in ulcerative colitis associated colorectal carcinoma: Upregulation of claudin-1, claudin-3, claudin-4, and β-catenin. Int. J. Colorectal Dis..

[42] De Oliveira S.S., de Oliveira I.M., De Souza W., Morgado-Díaz J.A. (2005). Claudins upregulation in human colorectal cancer. Febs Lett..

[43] Flasch M., Bueschl C., Woelflingseder L., Schwartz-Zimmermann H.E., Adam G., Schuhmacher R., Marko D., Warth B. (2020). Stable Isotope-Assisted Metabolomics for Deciphering Xenobiotic Metabolism in Mammalian Cell Culture. ACS Chem. Biol..

[44] Fruhmann P., Warth B., Hametner C., Berthiller F., Horkel E., Adam G., Sulyok M., Krska R., Fröhlich J. (2012). Synthesis of deoxynivalenol-3-ß-D-O-glucuronide for its use as biomarker for dietary deoxynivalenol exposure. World Mycotoxin J..

[45] Schmutz C., Cenk E., Marko D. (2019). The *Alternaria* Mycotoxin Alternariol Triggers the Immune Response of IL-1beta-stimulated, Differentiated Caco-2 Cells. Mol. Nutr. Food Res..

[46] Schothorst R.C., van Egmond H.P. (2004). Report from SCOOP task 3.2.10 “collection of occurrence data of *Fusarium* toxins in food and assessment of dietary intake by the population of EU member states”. Subtask: Trichothecenes. Toxicol. Lett..

[47] Del Favero G., Hohenbichler J., Mayer R.M., Rychlik M., Marko D. (2020). Mycotoxin Altertoxin II Induces Lipid Peroxidation Connecting Mitochondrial Stress Response to NF-κB Inhibition in THP-1 Macrophages. Chem. Res. Toxicol..

[48] Del Favero G., Woelflingseder L., Braun D., Puntscher H., Kutt M.L., Dellafiora L., Warth B., Pahlke G., Dall’Asta C., Adam G. (2018). Response of intestinal HT-29 cells to the trichothecene mycotoxin deoxynivalenol and its sulfated conjugates. Toxicol. Lett..

[49] Chou T.-C. (2006). Theoretical Basis, Experimental Design, and Computerized Simulation of Synergism and Antagonism in Drug Combination Studies. Pharmacol. Rev..

[50] Webb J. (1963). Effect of More Than One Inhibitor.

